# Chemiexcitation in Ex Vivo Porcine Skin Model

**DOI:** 10.1111/pcmr.70060

**Published:** 2025-10-14

**Authors:** Pavel Pospíšil, Vendula Paculová, Ankush Prasad, Michal Berecka

**Affiliations:** ^1^ Department of Biophysics Faculty of Science, Palacký University Olomouc Czech Republic

**Keywords:** electronically excited species, lipid peroxidation, melanin, triplet carbonyl

## Abstract

Chemiexcitation, the formation of electronically excited states via oxidative chemical reactions, has emerged as a potentially important contributor to skin photobiology beyond direct damage caused by ultraviolet (UV) radiation. This study investigates the hypothesis that UV radiation induces chemiexcitation in skin through the formation of triplet excited carbonyls, which may transfer energy to melanin and contribute to oxidative stress even after the termination of UV exposure. Using porcine skin as a model, we demonstrate that UV exposure leads to lipid peroxidation and the subsequent formation of organic radicals, including carbon‐centered (alkyl) and oxygen‐centered (peroxyl and alkoxyl) species, as detected by EPR spin‐trapping spectroscopy. HPLC‐MS analysis revealed that short‐chain carbonyl compounds, such as formaldehyde and acetaldehyde, are the predominant electronically excited species in direct chemiexcitation. These triplet carbonyls can transfer their excitation energy to melanin through photon emission (radiative transfer) or direct electron exchange (non‐radiative transfer), forming melanin‐based secondary excited states via indirect chemiexcitation. These findings highlight a novel, light‐independent mechanism of post‐UV exposure oxidative damage in the skin and suggest a possible role for chemiexcitation in processes such as photoaging and photocarcinogenesis.

## Graphical abstract

Formation of triplet excited carbonyl by direct chemiexcitation in the fibroblasts of the dermis and in the melanocytes and keratinocytes of the epidermis in the porcine skin, followed by excitation energy transfer from triplet excited carbonyl to melanin via radiative and non‐radiative pathways by indirect chemiexcitation.

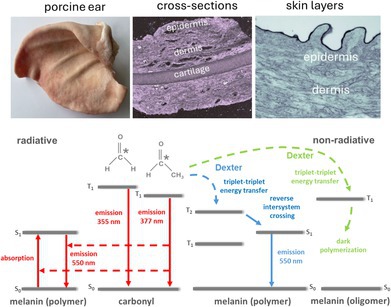

**Summary**

- UV radiation induces lipid peroxidation, forming reactive intermediates (dioxetane, tetroxide) that generate triplet carbonyls like formaldehyde and acetaldehyde.
- Triplet carbonyls transfer energy to melanin via radiative or non‐radiative pathways, producing excited melanin in melanocytes and keratinocytes.
- Direct and indirect chemiexcitation contribute to skin responses to UV, affecting photoprotection, photoaging, and DNA damage.

## 1 Introduction

The human skin is the largest organ of the body and serves as the primary barrier directly exposed to sunlight, the main natural source of ultraviolet (UV) radiation. While the upper atmosphere absorbs UV radiation with wavelengths below 295 nm, UVA radiation (315–400 nm) and the partially UVB band (280–315 nm) are absorbed mainly by the human skin. Exposure to UV radiation can have beneficial effects for stimulating the production of vitamin D as it promotes innate and adaptive immune responses (Bikle 2014). However, overexposure to UV radiation can lead to oxidative stress, which contributes to a range of deleterious effects (Sies et al. 2017) such as sunburn, premature skin aging, and various forms of skin cancer such as malignant melanoma (melanoma) and basal cell carcinoma (basalioma) (Ichihashi et al. 2003; Matsumura and Ananthaswamy 2004; Schuch et al. 2017). Under oxidative stress, key biomolecules, including lipids, proteins, and nucleic acids, undergo oxidative modifications, resulting in oxidative damage (lipid peroxidation, protein oxidation, nucleic acid oxidation) (Ichihashi et al. 2003; Niki 2009; Kammeyer and Luiten 2015; Davies 2016; Bernard et al. 2019; Di Mascio et al. 2019).

UV‐induced lipid peroxidation occurs through the absorption of UV radiation either directly by lipids (photoionization reaction) or indirectly by photosensitizers (photosensitization reaction) (Baptista et al. 2021; Alonso et al. 2009). In the photoionization reaction, the absorption of UVB radiation by unsaturated fatty acids leads to the formation of excited states (Bastos et al. 2023). The excited states undergo charge separation, resulting in the generation of cationic and anionic radicals (Schmalzbauer et al. 2021). In a photosensitization reaction, UVA radiation is absorbed by the photosensitizer, producing an excited photosensitizer which can either undergo charge separation to cationic and anionic radicals (Type I reaction) or transfer its excitation energy to molecular oxygen (Type II reaction). Both photoionization and photosensitization Type I reactions lead to the formation of reactive oxygen species (ROS) such as superoxide anion radical (O_2_^•−^), hydrogen peroxide (H_2_O_2_), and hydroxyl radical (HO^•^). Apart from the radical ROS, photosensitization Type II reaction leads to the formation of non‐radical ROS such as singlet oxygen (^1^O_2_) (Bickers and Athar 2006). Reactive oxygen species levels are regulated by both non‐enzymatic and enzymatic antioxidant systems (Stahl and Sies 2003; Masaki 2010; Dunaway et al. 2018; Martemucci et al. 2022). However, when the antioxidant system cannot eliminate undesirable ROS formation sufficiently, ROS (HO^•^ or ^1^O_2_) initiate lipid peroxidation (Niki 2015; Yin et al. 2011). During lipid peroxidation, carbon‐centered radicals such as alkyl radicals (R^•^) and oxygen‐centered radicals such as peroxyl (ROO^•^) and alkoxyl (RO^•^) radicals are formed. Cyclization or recombination of oxygen‐centered radicals (ROO^•^) forms reactive intermediates such as dioxetane and tetroxide, which further decompose to electronically excited species through a process known as chemiexcitation (Vacher et al. 2018; Ramos et al. 2023). In direct chemiexcitation, oxidation and excitation occur on the same molecule (Tzani et al. 2021; Cabello et al. 2023b). In indirect chemiexcitation, when oxidation and excitation occur on different molecules, the electronically excited molecule can transfer its excitation energy to another molecule, resulting in an excited state on the second molecule while the original returns to the ground state. The transition from the excited state to the ground state is accompanied by photon emission called ultra‐weak photon emission (Pospíšil et al. 2014; Van Wijk et al. 2020; Mould et al. 2024).

The formation of electronically excited states in biological processes has been under consideration for several decades (Vacher et al. 2018; Cilento and Adam 1995; Adam et al. 2005; Brash and Goncalves 2023; Cabello et al. 2023a; White et al. 1974; Adam and Cilento 1982; Bechara et al. 2017; Pospíšil et al. 2019). Significant progress in elucidating the mechanism of chemiexcitation has been achieved in chemical systems. Significant progress has been made in understanding the chemiexcitation mechanism involved in the decomposition of cyclic peroxides (1,2‐dioxetanes and 1,2‐dioxetanone) (Bastos et al. 2023; Cabello et al. 2023b; Augusto et al. 2017; Green et al. 2017). Over the past decade, technological advances have enabled the detection of photons emitted by cells, tissue, or whole organisms, allowing for the investigation of electronically excited state formation in more detail (Mano et al. 2014; Prasad and Pospíšil 2013). Brash and his colleagues demonstrated that melanin undergoes chemiexcitation under UVA exposure, and that energy transfer from excited melanin to DNA leads to the formation of cyclobutane pyrimidine dimers (CPDs) (Premi et al. 2015). It was shown that UVA radiation initiates the formation of nitric oxide (NO^•^) and (O_2_^•−^) catalyzed by nitric oxide synthase (NOS) and NADPH oxidase (NOX), respectively. The recombination of NO^•^ and O_2_^•−^ forms peroxynitrite (ONOO^−^), which oxidizes melanin to form a carbon‐centered radical. The carbon‐centered radical reacts with molecular oxygen to produce a peroxyl radical, which subsequently cyclizes to form an endoperoxide known as dioxetane. Thermal decomposition of the dioxetane generates a triplet excited carbonyl in melanin, which non‐radiatively transfers its excitation energy to DNA, resulting in the formation of CPDs between adjacent thymine or cytosine bases. Recent studies also revealed that chemiexcited neurotransmitters and hormones create CPD in the dark (Gonçalves et al. 2023). Moreover, the chemiexcitation process also plays a crucial role in photoreceptor disc turnover and the prevention of macular degeneration (Lyu et al. 2023).

To protect skin against UV‐induced lipid peroxidation, skin cells synthesize melanin, an essential skin pigment responsible for determining skin color (Song et al. 2023; Devaraj et al. 1997). Melanin synthesis, which occurs in specialized organelles of melanocytes called melanosomes, involves the enzymatic oxidation of L‐tyrosine to L‐DOPA and other oxidation products, which subsequently polymerize to form two main types of melanin: yellow‐reddish pheomelanin and brown‐black eumelanin (Cao et al. 2021). Pheomelanin acts as a photosensitizer, transferring excitation energy to molecular oxygen, forming ^1^O_2_ (Chiarelli‐Neto et al. 2014). It is predominantly produced in lighter skin types, particularly in response to UV exposure during the tanning process, thereby increasing the potential for photosensitization. In contrast, eumelanin does not act as a photosensitizer. Instead, it protects the skin against UV‐induced lipid peroxidation by effectively absorbing UV radiation (Brenner and Hearing 2008; Ortonne 2002; Meredith and Sarna 2006). The protective function is enabled by the unusually wide absorption spectrum of eumelanin, which spans UVB, UVA, and visible light (300–700 nm) (Nofsinger et al. 1999). While the UVB (280–315 nm) radiation penetrates only the upper layer of the skin (epidermis), the UVA radiation (315–400 nm) is capable of reaching the deeper layers of the skin (dermis) (Solano 2020). Melanocytes are localized in the basal layer of the epidermis, which is the deepest layer closest to the dermis. Within this layer, melanocytes are interspersed among basal keratinocytes, the primary type of cell in the basal layer.

In this study, the formation of electronically excited species was investigated after exposure to UV radiation from the porcine ear, whose histological structure is considered to be similar to that of human skin (Jacobi et al. 2007). Porcine and human skin share similarities in pigmentation, as well as in lipid and protein composition. Additionally, they exhibit comparable sizes, orientations, and blood vessel distribution, along with similar dermal to epidermal thickness ratios (Summerfield et al. 2015). We demonstrated that lipid peroxidation in the dark after UV radiation exposure is associated with the formation of triplet excited carbonyls ^3^(C=O)*, which can transfer excitation energy to melanin via radiative and non‐radiative pathways. Chemiexcitation of melanin may play a role in dark melanin synthesis and dark melanin polymerization, providing protection against damage to surrounding biomolecules.

## 2 Material and Methods

### 2.1 Sample Preparation

Fresh porcine ears were collected from a local slaughterhouse, transported on ice, and cleaned with saline solution (0.9% NaCl) and distilled water. Skin biopsies were prepared by small incisions from the inner side of the porcine ear using a surgical scalpel. The skin cross‐sections for the microscopic study were prepared from skin biopsies by cryogenic sectioning using the cryostat microtome set to a temperature of −20°C. The thickness of skin cross‐sections was set at 15 μm for optimal viewing. The skin cross‐sections were collected on a glass slide and stored at −20°C. To prepare skin homogenate for molecular study, skin biopsies, each weighing approximately 0.5 g, were cut into small pieces, frozen in liquid nitrogen, and homogenized in 4 mL of radioimmunoprecipitation assay (RIPA) buffer containing protease and phosphatase inhibitors. The homogenization was carried out using a rotor‐stator homogenizer, with three cycles of 30 s each, and the samples were cooled on ice after each cycle to prevent overheating. For the preparation of lipid extract, the skin homogenate was incubated at 300 μL of 35% HClO_4_ followed by centrifugation at 25,000 *g* for 10 min at 4°C to remove proteins by precipitation. For HPLC‐MS analysis, proteins were removed using Amicon Ultra Centrifugal Filter.

### 2.2 Sample Exposure to UV Radiation

For UV irradiation, skin samples (porcine ear, skin biopsy, skin homogenate, lipid extract) were placed in a petri dish and exposed to UV radiation generated by the LightningCure spotlight source LC8 using L8251 lamp (Hamamatsu Photonics K.K., Shizuoka, Japan). Radiation was conducted to the central area of the porcine ear (radiation spot area 1 cm^2^) or the biopsy (biopsy area 1 cm^2^) by light guide (the sample was positioned at 10 cm from the light guide). The irradiance on the surface of the skin was 14 W m^−2^. The corresponding UV doses for samples exposed to UV radiation for 0, 1, 3, 5, 7, and 10 min were 0.84, 2.5, 4.2, 5.9, and 8.4 kJ m^−2^. The petri dish with the sample was placed on ice to prevent heating of the skin surface produced by radiation. To determine the amount of radiation energy that fell on a porcine ear surface in spectral regions, the spectral irradiance of the UV/VIS lamp was measured (Figure S1A). The spectral irradiance shows dominant peaks in the UV range at around 320 and 370 nm and in the visible range at around 410 and 435 nm. The spectral characteristic of the lamp was measured using LI‐1800 Portable Spectroradiometer (LI‐COR, Lincoln, Nebraska, USA). For comparison, the spectral distribution of the lamp and the solar spectrum are shown side by side (Figure S2). This irradiation source emits within both the UVA and UVB ranges, which were not separately evaluated in this study. Based on spectral weighting, approximately 7 raw J/m^2^ from this lamp correspond to 1 erythemally weighted J/m^2^. The radiation source was located outside the experimental dark room.

### 2.3 Histological Characterization of Skin Cross‐Sections

Tissue sections were first air‐dried at room temperature. Slides were then immersed in Harris Hematoxylin solution (Sigma Aldrich GmbH, Germany) for 2 min to stain the nuclei. Excess stain was removed by rinsing the slides in running tap water. Differentiation was performed using a differentiation solution with two quick dips, followed again by rinsing in running tap water. To enhance nuclear contrast, slides were blued in Scott's tap water substitute for 60 s. Subsequently, the sections were dehydrated in 95% ethanol for 30 s. Cytoplasmic structures were stained by immersing the slides in Eosin Y solution (Sigma Aldrich GmbH, Germany) for 60 s, followed by a quick dip in distilled water. The stained tissue sections were examined using a NIB‐100 light microscope with a 20× objective lens.

### 2.4 Microscopic Characterization of Porcine Ear Biopsy

Microscopic imaging of skin cross‐sections was performed with a Bench Top bc43 spinning disk confocal microscope operated in brightfield mode (Andor Technology Ltd. Oxford Instruments, Belfast, Ireland). The microscope was controlled with Fusion image acquisition software (Benchtop version 1.101, Oxford Instruments) and processed with Imaris imaging software (version 10.0, Oxford Instruments).

### 2.5 Electron Paramagnetic Resonance Spin‐Trapping Spectroscopy

Electron paramagnetic resonance (EPR) spin‐trapping spectroscopy was used to measure the formation of carbon‐ and oxygen‐centered radicals in the skin extract isolated from the porcine ear. Carbon‐centered and oxygen‐centered radicals were detected using the hydrophilic spin traps 5‐(diethoxyphosphoryl)‐5‐methyl‐1‐pyrroline‐N‐oxide (DEPMPO) (Cayman Chemical, Ann Arbor, Michigan, USA). The skin extract was exposed to UV radiation in the presence of 50 mM DEPMPO and 40 mM PBS (pH 7.4). After UV exposure, the sample was immediately transferred into a glass capillary tube (Blaubrand intraMARK, Brand, Germany), and EPR spectra were collected at room temperature using an electron paramagnetic resonance spectrometer (MiniScope MS400, Magnettech GmbH, Berlin, Germany). The EPR conditions were as follows: microwave power, 10 mW; modulation amplitude, 0.1 mT; modulation frequency, 100 kHz; sweep width, 8.9 mT. Signal intensity was calculated from the height of the peak of the first derivative of the absorption. Simulation of EPR spectra was done using Winsim software, which is freely available from the website of the National Institute of Environmental Health Sciences (NIEHS 2002).

### 2.6 High‐Performance Liquid Chromatography

Carbonyls were identified and quantified by reverse‐phase high‐performance liquid chromatography (HPLC) using derivatization methods monitored by absorption spectroscopy (Fung and Grosjean 1981) and mass spectrometry (Grosjean et al. 1999) with some modifications. Derivatization of carbonyls was performed with 2,4‐dinitrophenylhydrazine (DNPH), forming hydrazone derivatives (formaldehyde‐DNPH and acetaldehyde‐DNPH derivatives). In this method, 1 μL of 50 mM DNPH dissolved in 50% sulfuric acid was added to the sample and incubated in the dark at 25°C for 30 min. Carbonyl‐DNPH derivatives were separated by isocratic elution with a Symmetry C18 (4.6 × 75 mm, 3.5 μm particle size) using an Alliance e 2695 HPLC System (Waters Corporation, Milford MA, USA). A mobile phase composed of 60% acetonitrile and 40% water flowed through the column at a rate of 0.6 mL min^−1^ at 25°C. A photodiode array (PDA) detector (Waters Corporation, Milford, MA, USA) was used to detect the carbonyl‐DNPH derivatives. The PDA detector was operated in a spectral range of 200–600 nm, and an optical resolution of 1.2 nm was used for detection. The evaluation of the carbonyl‐DNPH derivative was carried out at a wavelength of 360 nm. The calibration curves were determined using formaldehyde and acetaldehyde analytical standards (Sigma Aldrich GmbH, Germany). The QDa mass detector was operated in an electrospray negative ion mode by applying a voltage of 0.8 kV to the electrospray ionization interface capillary, and the cone voltage was set at 15 V. The desolvation temperature of the capillary probe was set at 600°C. A single ion chromatogram (SIR) of the peaks at the retention time 8.1 and 10.7 min was acquired using m/z 209 (formaldehyde‐DNPH derivative) and 223 (acetaldehyde‐DNPH derivative), respectively. Empower 3 software was used to evaluate the results obtained by HPLC analysis.

### 2.7 Ultra‐Weak Photon Emission

A highly sensitive charge‐coupled device (CCD) camera VersArray 1300B (Princeton Instruments, Trenton, NJ, USA) was used for the imaging of ultra‐weak photon emission. The dark current of the CCD camera was achieved by cooling it down to −110°C using a liquid‐nitrogen cooling system. The properties of the CCD camera are as follows: the spectral sensitivity of 350–1000 nm, the quantum efficiency of 90% in the visible range of the spectrum, 1340 × 1300 pixels. The following CCD camera parameters were used: scan rate, 100 kHz; gain, 2; and accumulation time of 20 min. The data correction was done by subtracting the background noise from every measurement. The CCD camera was situated in an experimental dark room with an interior painted black and protected from light entering the room from the outer dark room. To avoid any possible interference by external light, the data recording computer was installed in the outer dark room. The data collection from UV‐irradiated porcine skin was started 5 min after termination of irradiation. A low‐noise photomultiplier tube (PMT) R7518P and photon counting unit C9744 (Hamamatsu Photonics K.K., Iwata city, Japan) were used to detect the kinetics of ultra‐weak photon emission measured after 20 s of termination of irradiation. To reduce the thermal electrons, the PMT was cooled down to −30°C using thermoelectric cooler C9143 (Hamamatsu Photonics, K.K., Iwata city, Japan). The spectral sensitivity of the PMT was in the spectral range of 185–730 nm. The overall noise comprising dark count and background light was 2 counts s^−1^. The dark count was adjusted to approximately 1.5 counts s^−1^ at −1150 mV. The distance between the skin sample and the PMT window was kept at 2 cm.

## 3 Results

### 3.1 Microscopic Characterization of Ex Vivo Porcine Skin Model

**FIGURE 1:**
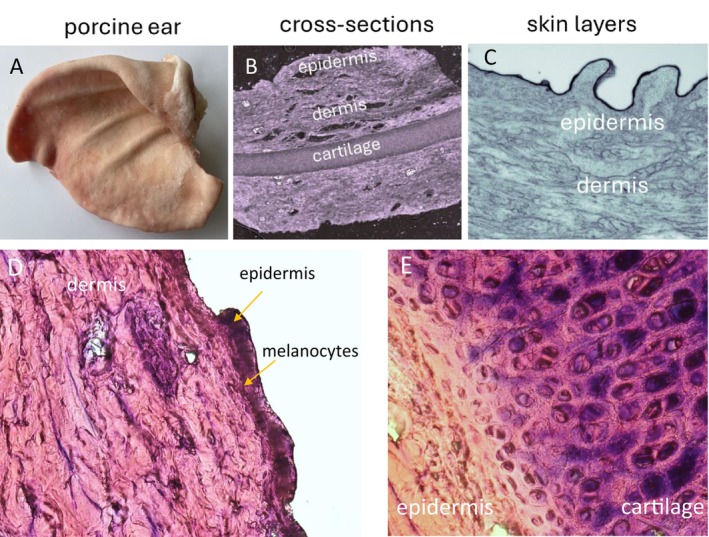
Microscopic characterization of porcine ear biopsy. (A) Photo of the porcine ear. The fresh porcine ears were obtained from a local slaughterhouse. (B) Cross‐section of porcine skin. Skin cross‐sections were prepared from skin biopsies by cryogenic sectioning with a thickness of 15 μm. (C) Skin layers of porcine cross sections. The layer of skin cross‐section contains the epidermis and dermis. (D) Histological image of porcine skin stained with hematoxylin and eosin, showing the epidermis and dermis. Arrows indicate the epidermis with localization of melanocytes in the basal layer of the epidermis. (E) Hematoxylin and eosin‐stained section showing the dermis and underlying cartilage.

Ex vivo data collection was conducted using fresh porcine ears or skin biopsies obtained from fresh porcine ears (Figure 1A). As in the ex vivo porcine skin model, the cell population (keratinocytes, melanocytes, and fibroblasts) and dermal matrix (collagen, elastin) are maintained; porcine ears or skin biopsies mimic in vivo human skin. Microscopic characterization of the skin biopsies was performed by imaging cross‐sections prepared from skin biopsies by cryogenic sectioning captured using a confocal microscope operated in brightfield mode (Figure 1B). A cross‐sectional view of the skin reveals the epidermal and dermal layers along with elastic connective tissue cartilage, which occupies the central part of the section. Detailed focus on skin layers shows the dermis with collagen and elastin fibers that contribute to skin strength and elasticity (Figure 1C). Hematoxylin and eosin staining provided a clear overview of the general skin tissue architecture, distinguishing the cartilage, dermis, and epidermis, with melanocytes typically localized in the basal layer of the epidermis (Figure 1D,E).

### 3.2 Detection of Carbon‐ and Oxygen‐Centered Radicals by EPR Spin‐Trapping Spectroscopy

**FIGURE 2:**
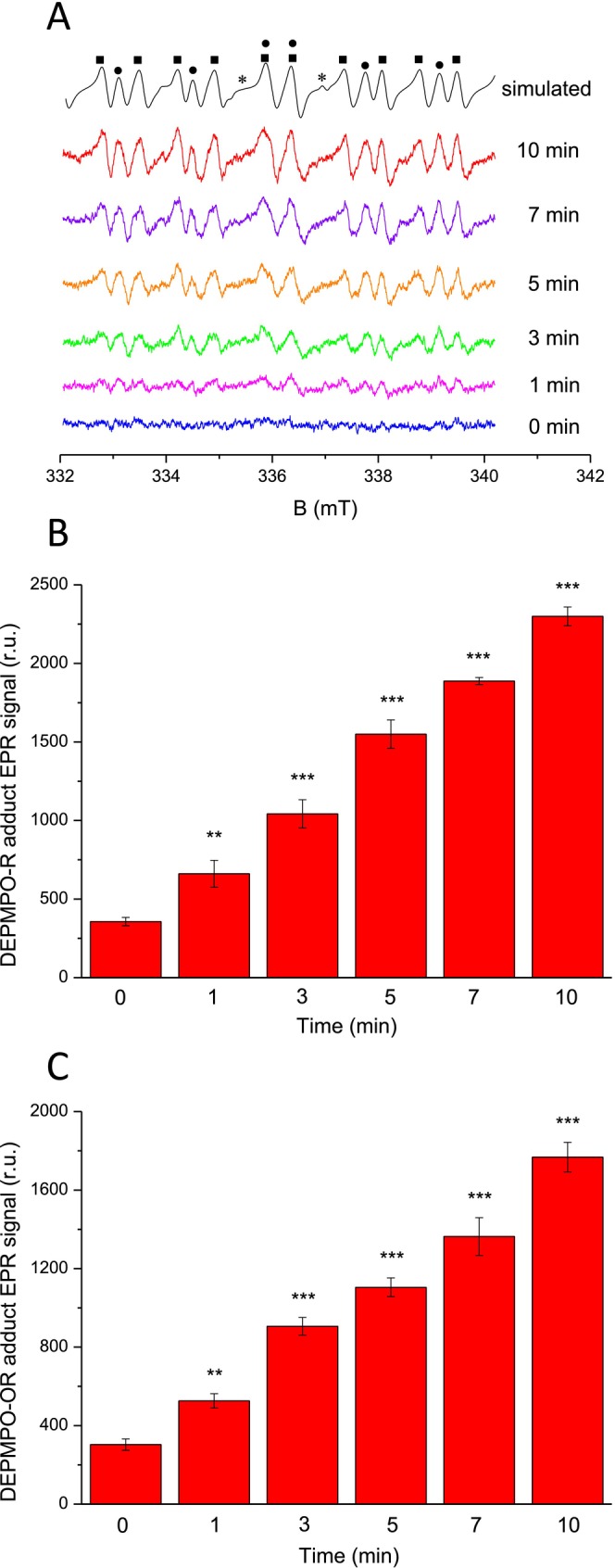
Detection of carbon‐ and oxygen‐centered radicals in skin extract by EPR spin‐trapping spectroscopy. (A) Carbon‐centered (R^•^) and oxygen‐centered (ROO^•^ and RO^•^) radicals monitored by DEPMPO‐R (square), DEPMPO‐OOR (star), and DEPMPO‐OR (circle) adduct EPR spectra. The skin extract was exposed to UV radiation for 0, 1, 3, 5, 7, and 10 min before measuring the EPR spectra. The EPR spectra were recorded in the presence of 50 mM DEPMPO, skin extract, and 25 mM PBS (pH 7.4). The simulated spectrum was obtained using three spectral components with hyperfine coupling constants (1) *a* ^N^ = 1.42 mT, *a* ^H^ = 2.11 mT, *a* ^P^ = 4.56, (2) *a* ^N^ = 1.14 mT, *a* ^H^ = 1.23 mT, *a* ^P^ = 5.31, and (3) *a* ^N^ = 1.39 mT, *a* ^H^ = 1.32 mT, *a* ^P^ = 4.65. (B, C) Time profile of DEPMPO‐R and DEPMPO‐OR adduct EPR signal. Signal intensity was evaluated as the relative height of the peak of the first derivative of the absorption for DEPMPO‐R (B) and DEPMPO‐OR (C) adduct. The bar graph represents the mean (*n* = 3, ± SD). Statistical analysis was performed using Student's t‐test of the difference between lipid extract treated with UV for 0 min and for 1, 3, 5, 7, and 10 min. Results were denoted as follows: *p* < 0.05 (*), *p* < 0.01 (**), *p* < 0.001 (***).

To monitor the formation of carbon‐ and oxygen‐centered radicals in skin extract isolated from porcine ear biopsy, the EPR spin‐trapping technique was employed using cyclic nitrone spin trap compound DEPMPO (Figure 2). The spin‐trapping of R^•^, ROO^•^, and RO^•^ forms DEPMPO‐R, DEPMPO‐OOR, and DEPMPO‐OR adducts. No EPR signal was detected when the spin trap was added to the skin extract kept in the dark. When skin extract was exposed to UV radiation, the DEPMPO‐R, DEPMPO‐OOR, and DEPMPO‐OR adduct EPR signals were detected (Figure 2A). The best simulation of experimental data was accomplished using three spectral components with hyperfine coupling constants: (1) *a* ^N^ = 1.42 mT, *a* ^H^ = 2.11 mT, *a* ^P^ = 4.56; (2) *a* ^N^ = 1.14 mT, *a* ^H^ = 1.23 mT, *a* ^P^ = 5.31; and (3) *a* ^N^ = 1.39 mT, *a* ^H^ = 1.32 mT, *a* ^P^ = 4.65, which are in good agreement with the hyperfine coupling constants attributed to DEPMPO‐R, DEPMPO‐OOR, and DEPMPO‐OR adducts (Karoui et al. 2011). The simulated spectrum was obtained considering 84%, 2%, and 14% contributions of DEPMPO‐R, DEPMPO‐OOR, and DEPMPO‐OR adduct EPR signal components. It was previously shown that the DEPMPO‐OOR adduct formed by the interaction of DEPMPO with ROO^•^ decomposes to RO^•^, which is subsequently trapped by DEPMPO, forming the DEPMPO‐OR adduct (Dikalov and Mason 2001). Based on these considerations, the detection of the DEPMPO‐OR adduct reflects the formation of ROO^•^ and RO^•^. When the height of DEPMPO‐R and DEPMPO‐OR adduct EPR signals was plotted against the period, the time profile showed gradual enhancement in the DEPMPO‐R (Figure 2B) and DEPMPO‐OR (Figure 2C) adduct EPR signals. Exposure of pure spin trap without skin extract to UV radiation does not induce any EPR signal during the whole period of UV irradiation. These results indicate that the exposure of skin to UV radiation is associated with the formation of carbon‐centered (R^•^) and oxygen‐centered (ROO^•^ and RO^•^) radicals.

### 3.3 Identification of Lipid Carbonyl by HPLC‐PDA/MS

**FIGURE 3:**
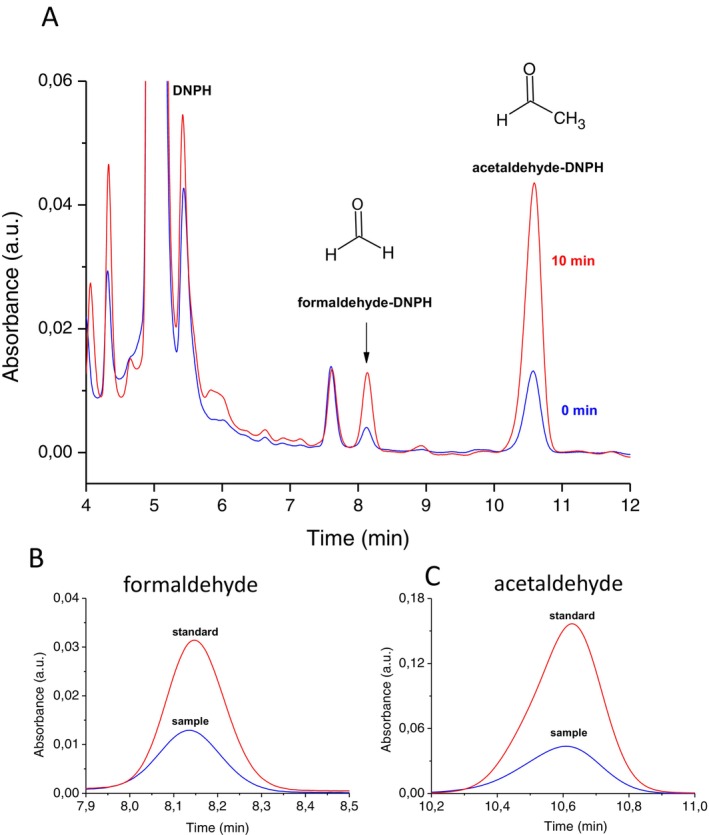
Identification of lipid carbonyl by HPLC‐PDA. (A) Chromatogram of formaldehyde and acetaldehyde‐DNPH derivatives in lipid extract isolated from porcine ear biopsy. The lipid extract was exposed to UV radiation for 0 and 10 min. The retention times of formaldehyde‐DNPH and acetaldehyde‐DNPH derivatives were 8.1 and 10.7 min, respectively. (B) The peak of the formaldehyde‐DNPH derivative measured in lipid extract, and the acetaldehyde standard added to the sample before derivations. (C) The peak of the acetaldehyde‐DNPH derivative measured in lipid extract and the acetaldehyde standard added to the sample before derivations.

**FIGURE 4:**
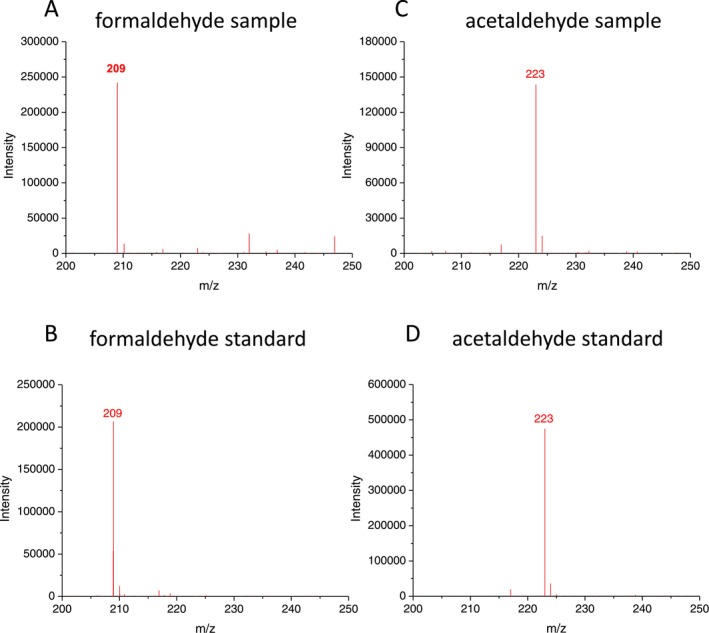
Identification of lipid carbonyl by HPLC‐MS. (A) The mass spectrum of the formaldehyde‐DNPH derivatives in lipid extract isolated from porcine ear biopsy. (B) The mass spectrum of the formaldehyde‐DNPH derivative measured with the acetaldehyde standard added to the sample before derivations. (C) The mass spectrum of the acetaldehyde‐DNPH derivatives in lipid extract isolated from porcine ear biopsy. (D) The mass spectrum of the acetaldehyde‐DNPH derivative measured with the acetaldehyde standard added to the sample before derivations.

To monitor lipid peroxidation in skin exposed to UV radiation, the carbonyls in lipid extract isolated from porcine ear biopsy were measured by reverse‐phase HPLC analysis using dinitrophenylhydrazine (DNPH) derivatizations. Hydrazone derivatives of carbonyls, formed by derivatization of carbonyls with DNPH, were eluted by isocratic separation on a reverse‐phase HPLC column and determined by absorbance at 360 nm. Figure 3A shows a chromatogram of the carbonyl‐DNPH derivative measured in UV‐exposed lipid extract. The carbonyl‐DNPH derivatives were eluted as follows: formaldehyde‐DNPH derivative (8.1 min) and acetaldehyde‐DNPH derivative (10.7 min). To confirm the formaldehyde‐DNPH and acetaldehyde‐DNPH derivatives, formaldehyde and acetaldehyde standards were added to the sample prior to derivatization (Figure 3B,C). A comparison of the carbonyl‐DNPH derivatives measured in the lipid extract and the standard indicates that the peaks observed at 8.1 and 10.7 min could be assigned to the formaldehyde‐DNPH and acetaldehyde‐DNPH derivatives, respectively. To verify the origin of carbonyl‐DNPH derivatives, mass spectrometry determination of carbonyls was performed using a QDa mass detector. Figure 4A–D shows mass spectra obtained in the range of 200 to 250 mass‐to‐charge ratio (m/z) of charged particles (ions) measured in lipid extract (panel A and C) and standards (panels B and D). Mass spectrometry identification of formaldehyde and acetaldehyde accomplished using a QDa mass detector showed the m/z ratios 209 and 223, respectively, which correspond well to the value presented in the literature (Miller et al. 2010). These results confirm that the peaks observed at 8.1 and 10.7 min measured in skin lipid extract correspond to the formaldehyde‐DNPH and acetaldehyde‐DNPH derivatives.

### 3.4 Quantification of Lipid Carbonyl by HPLC‐PDA

**FIGURE 5:**
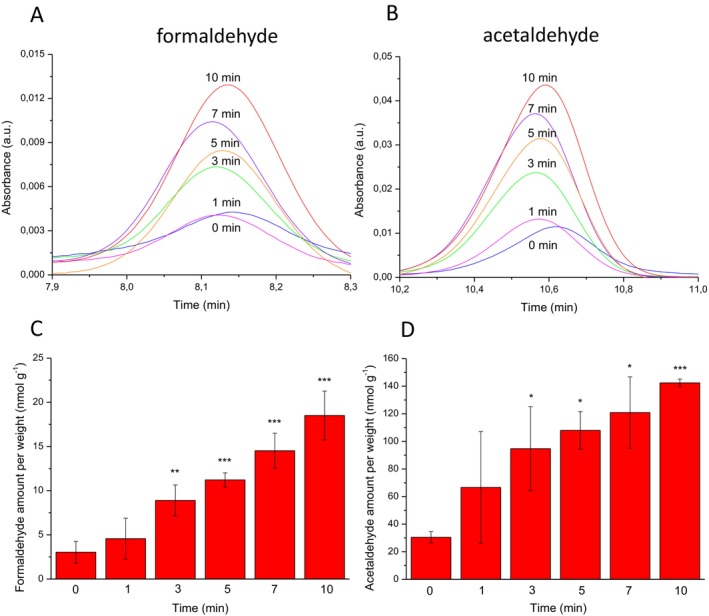
Quantification of lipid carbonyl by HPLC‐PDA. The peak of the formaldehyde‐DNPH (A) and acetaldehyde‐DNPH (B) derivatives measured in lipid extract exposed to UV radiation at different times. Time profile of formaldehyde (C) and acetaldehyde (D) amount formed in lipid extract. Formaldehyde and acetaldehyde amount were determined as the area under a peak at the retention time of 8.1 and 10.7 min using the calibration curve obtained using the formaldehyde and acetaldehyde standards. The calibration curve was constructed by plotting the peak area versus the concentration of standard compounds. The calibration curve showed good linearity with a coefficient of determination of 1.0 (formaldehyde) and 0.998 (acetaldehyde) within the test range. The amount of carbonyls is expressed per 1 g of porcine ear biopsy used for isolation of lipid extract. The bar graph represents the mean (*n* = 3, ± SD). Statistical analysis was performed using Student's *t*‐test of the difference between lipid extract treated with UV for 0 min and for 1, 3, 5, 7, and 10 min. Results were denoted as follows: *p* < 0.05 (*), *p* < 0.01 (**), *p* < 0.001 (***).

Figure 5 shows the peak of the carbonyl‐DNPH derivative measured in a lipid extract isolated from porcine ear biopsy subjected to 0‐, 1‐, 3‐, 5‐, 7‐, and 10‐min UV radiation. The peak of the carbonyl‐DNPH derivative measured in lipid extract exposed to UV radiation for 0 min is likely formed by the effect of the derivatization agent dissolved in 50% sulfuric acid on the walls of the polypropylene Eppendorf tubes. The peak area of the UV‐exposed lipid extract after 10‐min UV radiation was four times higher than that of the control sample (no radiation) (Figure 5A,B). The areas under the peak were recalculated to carbonyl amounts using the calibration curve of the formaldehyde and acetaldehyde standards. The amount of formaldehyde and acetaldehyde was in the range of units and tens of nmol per 1 g of porcine ear biopsy used for the isolation of lipid extract (Figure 5C,D). These results indicate that the exposure of lipid extract to UV radiation caused the formation of formaldehyde and acetaldehyde, which gradually increased with increasing UV exposure time.

### 3.5 Electronically Excited Species Monitored by Ultra‐Weak Photon Emission

**FIGURE 6:**
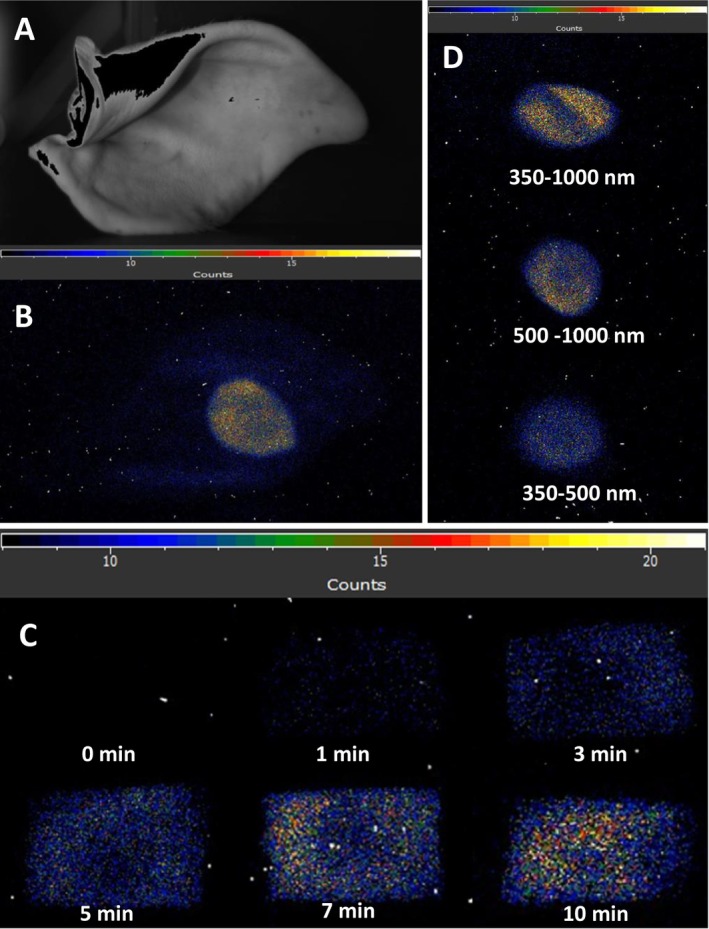
Detection of electronically excited species measured by ultra‐weak photon using CCD camera (A) Photographs of the porcine ear. (B) Ultra‐weak photon emission measured in the porcine ear biopsy exposed to UV radiation for 10 min. (C) Time dependence of ultra‐weak photon emission measured in the porcine ear biopsy after 0, 1, 3, 5, 7, and 10 min of UV irradiation. (D) Spectral property of ultra‐weak photon emission.

To monitor the formation of electronically excited species, two‐dimensional imaging of ultra‐weak photon emission was measured after exposure of the porcine ear to UV radiation using a CCD camera (Figure 6). The porcine ear (Figure 6A) was exposed to UV radiation conducted to the skin by a light guide (radiation spot size 1 cm^2^) and subsequently kept in the dark for 5 min to avoid delayed luminescence. The porcine ear exhibits spontaneous ultra‐weak photon emission in the entire skin ear and UV radiation‐induced ultra‐weak photon emission localized at the irradiated spot (Figure 6B). To test the time dependence, ultra‐weak photon emission was measured from the porcine ear biopsy after different periods of UV irradiation. Figure 6C shows that ultra‐weak photon emission increases with increasing time of UV exposure. The two‐dimensional imaging of ultra‐weak photon emission reveals that electronically excited species are formed spontaneously in the porcine ear, and the formation of electronically excited species is significantly enhanced at the irradiated spot of the porcine ear.

### 3.6 Spectral Property of Ultra‐Weak Photon Emission

To study the spectral property of the ultra‐weak photon emission, the photon emission from the UV radiation‐exposed porcine ear was determined using edge filters (Figure 6D). When detection of ultra‐weak photon emission was prevented in the blue region of the spectrum using a long‐pass edge filter passing wavelengths above 500 nm (Figure S1B), the photon emission measured in the spectral range of 500–1000 nm was partially decreased compared to the photon emission measured in the whole spectral range of 350–1000 nm without a filter. To limit the detection of ultra‐weak photon emission for the spectral range 350–500 nm, the combination of a long‐pass edge filter passing wavelengths above 350 nm and a short‐pass edge filter passing wavelengths above 500 nm was used (Figure S1C). When detection of ultra‐weak photon emission was prevented in the green‐red region of the spectrum, the photon emission measured in the spectral range of 350–500 nm was considerably decreased compared to the photon emission measured in the whole spectral range of 350–1000 nm without a filter. These results reveal that electronically excited species that emit photons in the blue region of the spectrum partially contribute to the overall photon emission, whereas electronically excited species emitting in the green‐red region of the spectrum produce most of the photons.

### 3.7 Kinetic Property of Ultra‐Weak Photon Emission

**FIGURE 7:**
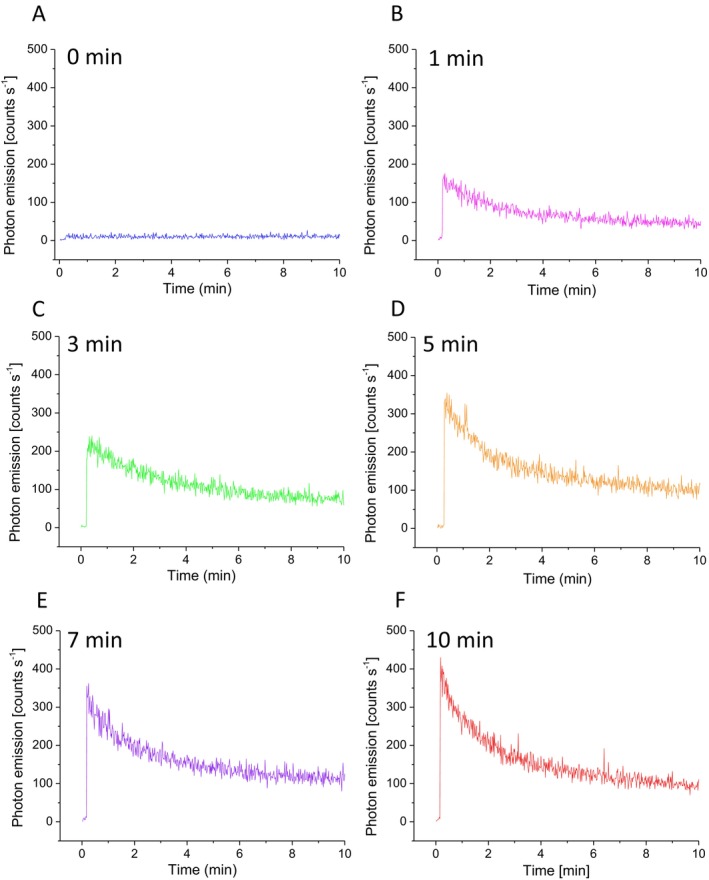
Kinetics of formation of electronically excited species measured by ultra‐weak photon using PMT. Time dependence of ultra‐weak photon emission measured in the porcine ear biopsy after 0, 1, 3, 5, 7, and 10 min of UV irradiation. After exposure to UV radiation, the porcine ear biopsy was placed in 20 s under the PMT window and subjected to the detection of ultra‐weak photon emission.

To study the kinetic properties of ultra‐weak photon emission, one‐dimensional ultra‐weak photon emission was measured after exposure of porcine ear biopsy to UV radiation using PMT (Figure 7). In the non‐exposed sample, the ultra‐weak photon emission was observed to remain constant in count rate over a measured period of 10 min. After subtracting the dark count of the PMT, the spontaneous ultra‐weak photon emission was determined to be 5 counts s^−1^. After exposure to UV radiation, the porcine ear biopsy was placed under the PMT window after 20 s and subjected to the detection of ultra‐weak photon emission. Ultra‐weak photon emission gradually decreases after switching off UV radiation and approaches the steady‐state value. The photon emission immediately after exposure end was determined to be several hundred counts s^−1^, decreasing to a value below 100 counts s^−1^ at the measured period of 10 min. Kinetic analysis shows that electronically excited species are formed after exposure to UV, and their formation gradually decreases over time.

### 3.8 Effect of Exogenous Melanin on Ultra‐Weak Photon Emission

**FIGURE 8:**
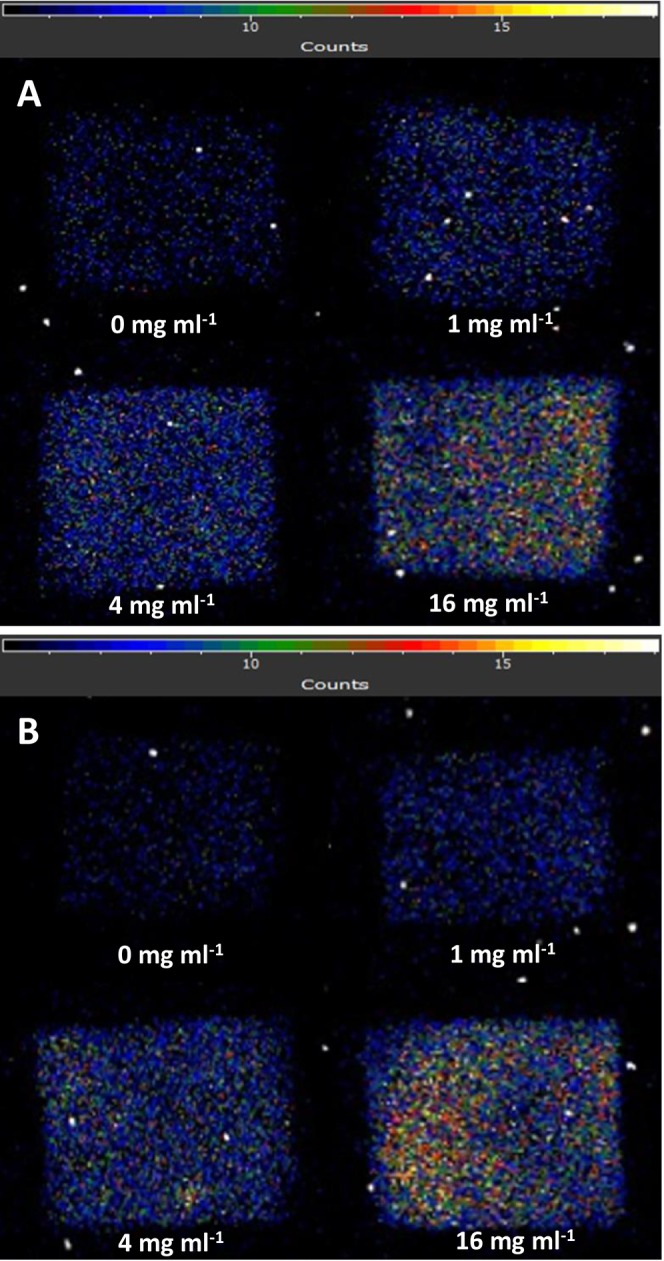
Effect of exogenous melanin on the formation of electronically excited species measured by ultra‐weak photon using CCD camera (A) Effect of exogenous melanin on ultra‐weak photon emission from porcine skin biopsy exposed to UV radiation for a different time. (B) Effect of exogenous melanin on ultra‐weak photon emission from porcine skin biopsy exposed to UV radiation for different times measured with a separating slide.

To test the involvement of melanin in the ultra‐weak photon emission, melanin was applied directly to the surface of porcine ear biopsy after exposure to UV radiation. Two‐dimensional images of ultra‐weak photon emission of porcine ear biopsy with melanin applied directly on the skin biopsy after exposure to UV radiation are shown in Figure 8A. When melanin was added directly to the surface of the skin biopsy, the ultra‐weak photon emission was enhanced with increasing mass concentration (0, 1, 4, and 16 μg ml^−1^). To prevent direct contact between the skin biopsy and melanin, melanin was placed between two coverslips and placed on the skin biopsy. Figure 8B shows two‐dimensional images of ultra‐weak photon emission of porcine ear biopsy with melanin applied on the skin biopsy after exposure to UV radiation. When melanin was separated from the surface of a skin biopsy by the glass, the ultra‐weak photon emission was enhanced similarly to direct contact.

## 4 Discussion

In this study, we present evidence that electronically excited species are formed as a result of lipid peroxidation, producing short‐chain carbonyls (formaldehyde and acetaldehyde), which can transfer excitation energy to melanin. UV radiation‐induced lipid peroxidation is associated with the formation of high‐energy reactive intermediates such as dioxetane and tetroxide. The decomposition of reactive intermediates leads to the formation of ^3^(C=O)* both in the fibroblasts of the dermis and in the melanocytes and keratinocytes of the epidermis (direct chemiexcitation). The transfer of excitation energy from ^3^(C=O)* to melanin via non‐radiative and radiative manners results in the formation of excited melanin, a pigment predominantly found in the epidermis, within melanocytes and keratinocytes (indirect chemiexcitation).

### 4.1 Direct and Indirect Oxidation of Lipids by UV Radiation

**FIGURE 9:**
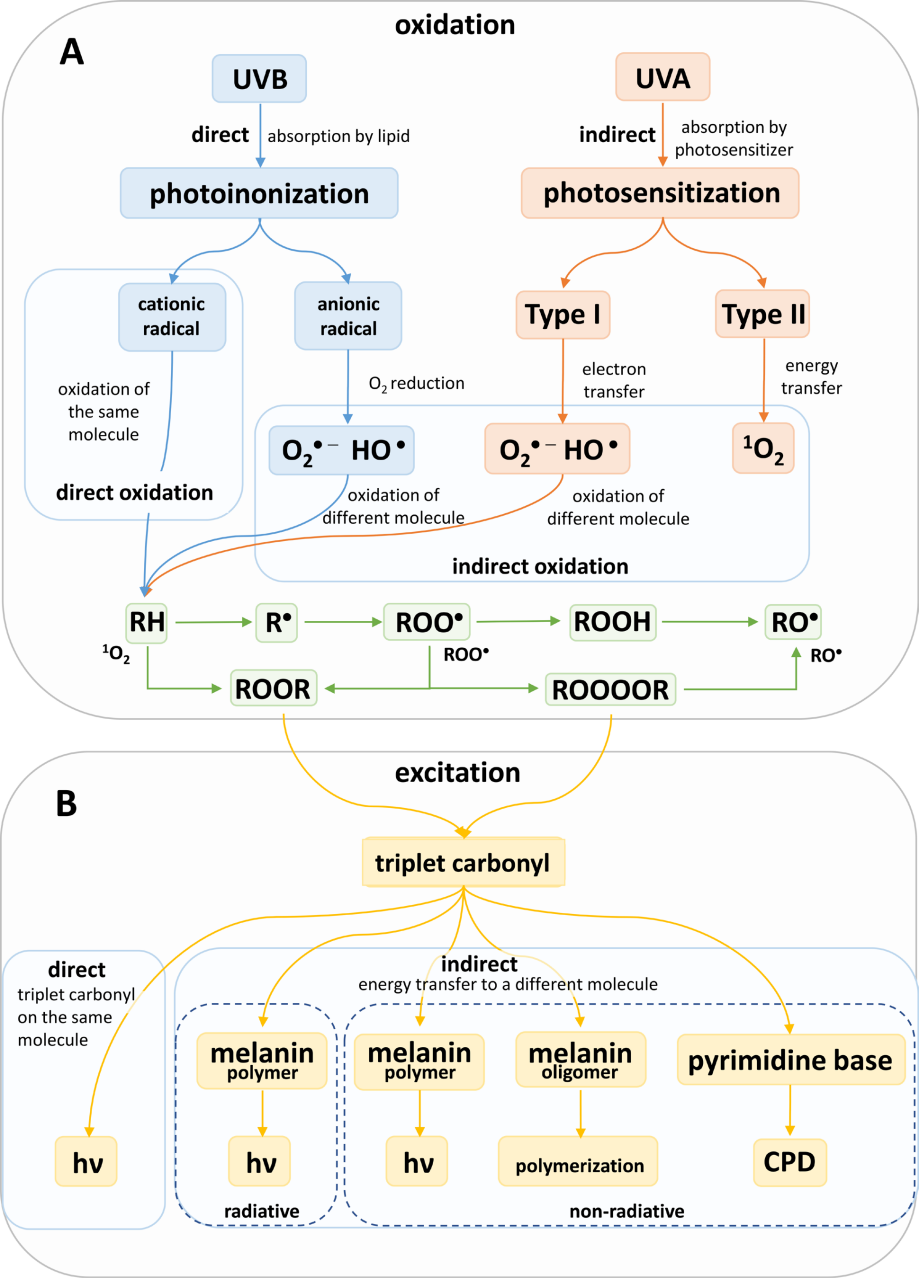
Direct and indirect chemiexcitation by UV radiation. (A) UVB radiation is directly absorbed by unsaturated fatty acids, generating an excited state that undergoes charge separation via photoionization, producing cationic and anionic radicals. The anionic radicals reduce molecular oxygen to form radical reactive oxygen species, such as superoxide anion and hydroxyl radicals. UVA radiation is absorbed by photosensitizers, which transition to a triplet excited state via intersystem crossing. Triplet photosensitizers then induce reactive oxygen species formation through Type I (electron transfer, generating superoxide anion and hydroxyl radicals) or Type II (energy transfer, generating singlet oxygen) reactions. Both radical and non‐radical reactive oxygen species initiate lipid peroxidation. In the radical pathway, hydroxyl radical abstracts hydrogen atoms from polyunsaturated fatty acids, leading to the formation of alkyl radical, peroxyl radical, hydroperoxide and alkoxyl radical. In the non‐radical pathway, singlet oxygen reacts with polyunsaturated fatty acids to form endoperoxides via cycloaddition. Recombination or cyclization of peroxyl radical gives rise to reactive intermediates (dioxetanes and tetroxides). (B) Reactive intermediates undergo thermal or chemical decomposition to form triplet carbonyl. In direct excitation, triplet carbonyls relax to the singlet ground state. In indirect excitation, the triplet excitation energy can be transferred to nearby melanin or DNA. In radiative reaction, excitation energy of triplet carbonyls is transferred to melanin, which emits a photon. In non‐radiative reaction, excitation energy is transferred to melanin (polymer or oligomer) via an electron exchange mechanism. The excited melanin polymer emits a photon, whereas the melanin oligomer uses the excitation energy for oxidative polymerization. In addition, non‐radiative energy transfer to DNA can induce the formation of cyclobutane pyrimidine dimers in the absence of ultraviolet light.

The direct absorption of UVB radiation by unsaturated fatty acids causes the formation of an excited state of the unsaturated fatty acid, which undergoes a charge separation, forming cationic and anionic radicals via photoionization reactions (Figure 9A). The anionic radicals can reduce molecular oxygen, forming radical ROS (O_2_^•−^ and HO^•^). The absorption of UVA by a photosensitizer causes a transition from the ground state to the singlet excited state, which is later converted to the triplet excited state via intersystem crossing (Elliott et al. 2020). The triplet‐excited photosensitizers undergo either Type I (electron transfer) or Type II (excitation energy transfer) reactions, both of which lead to ROS formation (Baptista et al. 2017). In the Type I reaction, charge separation of triplet excited photosensitizers forms cationic and anionic radicals, which in the presence of molecular oxygen, generate radical ROS (O_2_^•−^ and HO^•^). In the Type II reaction, energy transfer from the triplet excited photosensitizer to molecular oxygen produces non‐radical ROS (^1^O_2_). Lipid peroxidation is initiated both by radical ROS (HO^•^) and non‐radical ROS (^1^O_2_) (Niki 2015). In the radical pathway, abstraction of a hydrogen atom from a carbon atom of polyunsaturated fatty acids by HO^•^ leads to the formation of carbon‐centered (R^•^) and oxygen‐centered (ROO^•^ and RO^•^) radicals (Herrling et al. 2003). Detection of organic radicals using EPR spin‐trapping spectroscopy shows that the exposure of skin to UV radiation is associated with the formation of R^•^, ROO^•^, and RO^•^ (Figure 2). In the non‐radical pathway, ^1^O_2_ forms endoperoxides and hydroperoxides (Di Mascio et al. 2019). Endoperoxides are generated by cycloaddition of ^1^O_2_ to polyunsaturated fatty acids, while lipid hydroperoxides are produced via the ene reaction with polyunsaturated fatty. When ROO^•^ undergoes recombination or cyclization, reactive intermediates such as dioxetane and tetroxide are formed.

### 4.2 Direct Chemiexcitation of Lipids by Decomposition of High‐Energy Intermediates

**FIGURE 10:**
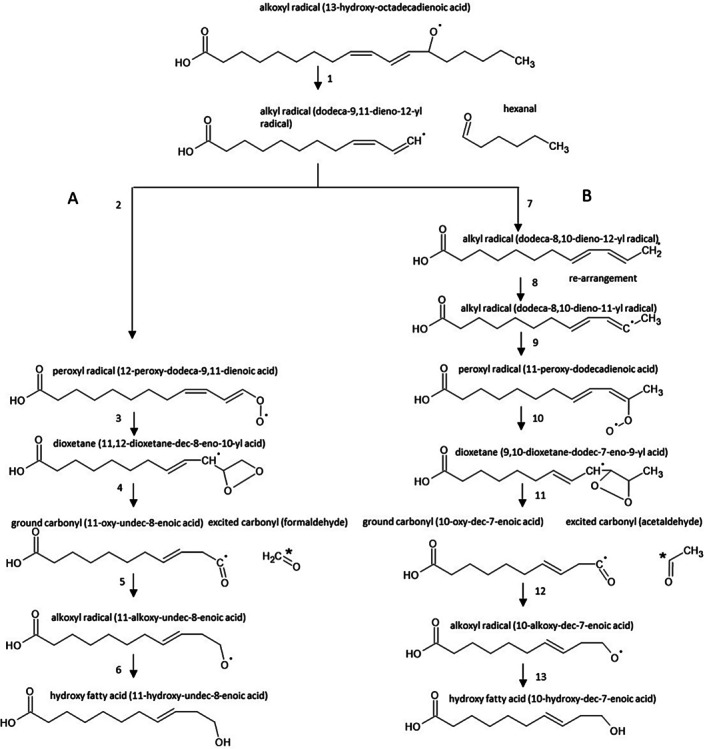
Formaldehyde and acetaldehyde formation by dioxetane decomposition. β‐scission of alkoxyl radical (13‐hydroxy‐octadecadienoic acid) forms alkyl radical (dodeca‐9,11‐dieno‐12‐yl radical) and carbonyl (hexanal) (reaction 1). (A) Formaldehyde formation. Oxidation of alkyl radical (dodeca‐9,11‐dieno‐12‐yl radical) results in the formation of peroxyl radical (12‐peroxy‐dodeca‐9,11‐dienoic acid) (reaction 2). The cyclization of peroxyl radical forms dioxetane (11,12‐dioxetane‐dec‐8‐eno‐10‐yl acid) (reaction 3), which decomposes to ground carbonyl (11‐oxy‐undec‐8‐enoic acid) and triplet excited carbonyl (formaldehyde) (reaction 4). The rearrangement of ground carbonyl results in the formation of alkoxyl radical (11‐alkoxy‐undec‐8‐enoic acid) (reaction 5), which is reduced to hydroxy fatty acid (11‐hydroxy‐undec‐8‐enoic acid) (reaction 6). (B) Acetaldehyde formation. The rearrangement of alkyl radical (dodeca‐9,11‐dieno‐12‐yl radical) forms alkyl radical (dodeca‐8,10‐dieno‐12‐yl radical) (reaction 7) and subsequently alkyl radical (dodeca‐8,10‐dieno‐11‐yl radical) (reaction 8). The oxidation of alkyl radical (dodeca‐8,10‐dieno‐11‐yl radical) forms peroxyl radical (11‐peroxy‐dodecadienoic acid) (reaction 9). The cyclization of peroxyl radical forms dioxetane (9,10‐dioxetane‐dodec‐7‐eno‐9‐yl acid) (reaction 10), which decomposes to ground carbonyl (10‐oxy‐dec‐7‐enoic acid) and triplet excited carbonyl (acetaldehyde) (reaction 11). The rearrangement of ground carbonyl forms alkoxyl radical (10‐alkoxy‐dec‐7‐enoic acid) (reaction 12), which is reduced to hydroxy fatty acid (10‐hydroxy‐dec‐7‐enoic acid) (reaction 13).

The reactive intermediates (dioxetane, tetroxide) undergo to ^3^(C=O)* by thermal or chemical decomposition (direct chemiexcitation) (Ramos et al. 2023) (Figure 9B). Identification of carbonyls by HPLC‐MS analysis indicates that aliphatic carbonyls are the main electronically excited species formed via direct chemiexcitation (Figures 3 and 4). Short‐chain aliphatic carbonyls, comprising a short aliphatic carbon chain and a carbonyl group, such as formaldehyde (1‐carbon) and acetaldehyde (2‐carbon), increase with prolonged UV exposure (Figure 5). Figure 10 shows the formation of formaldehyde and acetaldehyde via dioxetane decomposition. β‐scission of the alkoxyl radical forms an alkyl radical (dodeca‐9,11‐dieno‐12‐yl radical) and a carbonyl (hexanal) (Figure 10, reaction 1). In formaldehyde formation, the interaction of the alkyl radical with molecular oxygen generates a peroxyl radical (Figure 10, reaction 2), which cyclizes to form 11,12‐dioxetane (Figure 10, reaction 3). The decomposition of 11,12‐dioxetane produces both ground carbonyl and triplet excited carbonyl (formaldehyde) (Figure 10, reaction 4). The rearrangement of ground carbonyl results in the formation of alkoxyl radical (11‐alkoxy‐undec‐8‐enoic acid) (Figure 10, reaction 5), which is reduced to hydroxy fatty acid (11‐hydroxy‐undec‐8‐enoic acid) (Figure 10, reaction 6). In acetaldehyde formation, the rearrangement of alkyl radical (Figure 10, reaction 7–8) and its subsequent reaction with molecular oxygen forms peroxyl radical (Figure 10, reaction 9), which cyclizes to form 9,10‐dioxetane (Figure 10, reaction 10). The decomposition of 9,10‐dioxetane forms ground carbonyl and triplet excited carbonyl (acetaldehyde) (Figure 10, reaction 11). The rearrangement of ground carbonyl forms alkoxyl radical (10‐alkoxy‐dec‐7‐enoic acid) (Figure 10, reaction 12), which is reduced to hydroxy fatty acid (10‐hydroxy‐dec‐7‐enoic acid) (Figure 10, reaction 13). During dioxetane decomposition, the energy stored in the strained ring leads to the transition of one electron from a carbonyl double bond to a higher energy level, along with the change in the spin orientation. The transition from the triplet excited state to the singlet ground state is accompanied by photon emission in the spectral range from near UVA to the green region (350–500 nm). Due to its triplet state, this transition is spin‐forbidden under quantum mechanical rules, resulting in a prolonged lifetime of the excited state. Fibroblasts, located in the dermis, are primarily exposed to UVA radiation, which penetrates beyond the basal epidermis and reaches the upper part of the dermis. In fibroblast cells, ^3^(C=O)* are formed via UVA‐induced photosensitization by endogenous chromophores (e.g., porphyrins, flavins, NADH). The epidermis is exposed to both UVB, which is absorbed in the upper layers, and UVA, which penetrates to the basal layer where melanocytes and keratinocytes reside. UVB can induce photoionization in melanocytes and keratinocytes, which results in the formation of ^3^(C=O)*, while UVA triggers photosensitization, leading to ^3^(C=O)* formation in both cell types.

### 4.3 Indirect Chemiexcitation of Melanin by Excitation Energy Transfer From Lipids

**FIGURE 11:**
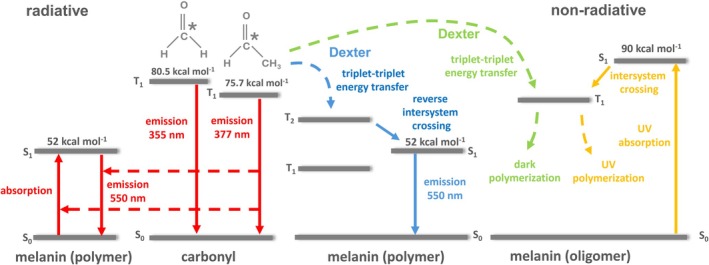
Schematic energy level diagram of excitation energy transfer from triplet excited carbonyl to melanin by radiative and non‐radiative pathways. In radiative energy transfer, the electronic transition from the triplet energy level (T_1_) of formaldehyde (80.5 kcal mol^−1^) or acetaldehyde (75.7 kcal mol^−1^) to the ground state is accompanied by photon emission at 355 nm and 377 nm, respectively. The photons emitted by triplet excited formaldehyde and acetaldehyde are absorbed by melanin (polymer), which emits photons at 550 nm. In the non‐radiative energy transfer, triplet‐triplet energy transfer via the Dexter mechanism from the first triplet energy level (T_1_) of formaldehyde (80.5 kcal mol^−1^) or acetaldehyde (75.7 kcal mol^−1^) proceeds either to the second triplet energy level (T_2_) of melanin (polymer) or the first triplet energy level (T_1_) of melanin (oligomer). In the polymeric form of melanin, the second triplet excited energy level (T_2_) undergoes the first excited energy level (S_1_) of melanin (52 kcal mol^−1^) by reverse intersystem crossing. The electronic transition from the first excited energy level (S_1_) to the ground state is accompanied by photon emission at 550 nm. In the oligomeric form of melanin, the excitation energy of the first triplet state (T_1_) might be used in dark melanin synthesis and dark melanin polymerization.

The long‐lived ^3^(C=O)* can transfer excitation energy to nearby melanin (Figure 9B). The triplet‐singlet energy transfer from ^3^(C=O)* to melanin is thermodynamically feasible as the triplet state of carbonyls (formaldehyde 80.5 kcal mol^−1^, acetaldehyde 75.7 kcal mol^−1^) is located above the singlet state of melanin (52 kcal mol^−1^). The excitation energy transfer proceeds through radiative and non‐radiative mechanisms (Figure 11). In the radiative reaction, a photon emitted by ^3^(C=O)* is absorbed by melanin. The energy of ^3^(C=O)* varies in the range of 80–60 kcal mol^−1^, which corresponds to the emission wavelength of 350–500 nm (Pospíšil et al. 2019). The triplet excited state of short‐chain aliphatic carbonyls exhibits the highest energy with emission wavelength in the UVA region: formaldehyde 355 nm (80.5 kcal mol^−1^) and acetaldehyde 377 nm (75.7 kcal mol^−1^) (Firme et al. 2013). The absorption spectrum of melanin spans a broad range from 300 to 700 nm with a distinguishable peak around 335 nm, followed by an exponential decay profile to the red region (Nofsinger et al. 1999). Such an absorption spectrum is a sum of absorption spectra for individual chromophore components within melanin (Wang and Blancafort 2021). The singlet excited melanin passes over to the ground state, emitting a photon at 550 nm. The emission wavelength depends on the number of different individual chromophores within the melanin polymer. Triplet carbonyls transfer excitation energy to melanin localized in melanocytes and keratinocytes in the basal layer of the epidermis, as well as in differentiated keratinocytes in the upper layers of the epidermis. In the non‐radiative reaction, energy transfer occurs via the Dexter mechanism. Dexter exchange energy transfer, which occurs at short range (< 10 Å) distance, is based on electron exchange, transferring the high‐energy electron of the donor for a ground state electron of the acceptor. As the Dexter mechanism is based on the spin conservation rule, the spin‐allowed processes are singlet‐singlet and triplet‐triplet energy transfer. A high‐energy excited electron from ^3^(C=O)* is transferred to nearby melanin, while a low‐energy electron from melanin is transferred to the carbonyl. In the melanin polymer, the triplet‐triplet energy transfer from the first triplet energy level of carbonyls (T_1_) occurs to the second triplet energy level of melanin (T_2_). A reverse intersystem crossing from the second triplet excited energy level (T_2_) to the first excited energy level of melanin (S_1_) follows. In the melanin oligomer, the triplet‐triplet energy transfer takes place from the first triplet energy level of carbonyls (T1) to the first triplet energy level of melanin (T1). According to the short‐range Dexter energy transfer mechanism, energy is transferred either within a single cell or between adjacent cells. In both melanocytes and keratinocytes, melanin‐containing melanosomes are often positioned within close proximity to lipid‐rich structures such as the plasma membrane and the nuclear envelope. In keratinocytes, supranuclear melanin caps are closely associated with the nuclear membrane, whereas melanosomes trafficked to or from the cell surface may transiently approach the plasma membrane, placing them in tight spatial proximity with lipid environments. The triplet excitation energy might be used for dark melanin polymerization. In dark polymerization, melanin oligomers form a larger polymer structure in oxidative reactions, where radicals facilitate the cross‐linking of melanin oligomers. The triplet carbonyl energy can be transferred to DNA forming dark cyclobutane pyrimidine dimers (Premi et al. 2015) which might upregulate gene expression for melanin synthesis (Brash et al. 1996).

## 5 Conclusion

The formation of electronically excited states in biological processes has been a subject of extensive research, with significant advancements in understanding the chemiexcitation mechanisms, particularly in the decomposition of cyclic peroxides. This study highlights the key finding that short‐chain carbonyls, formaldehyde, and acetaldehyde are the main electronically excited species formed via direct chemiexcitation. The transfer of excitation energy from ^3^(C=O)* to melanin, both non‐radiatively and radiatively, results in an excited state of melanin through indirect chemiexcitation. The study highlights the importance of electronically excited species in skin responses to UV radiation, offering potential pathways for developing protective strategies against photoaging and skin cancer.

## Author Contributions

Conceptualization, P.P. and V.P.; methodology, P.P. and V.P., A.P.; investigation, P.P., V.P., A.P., and M.B.; visualization, P.P. and V.P.; supervision, P.P.; writing – original draft, P.P. and V.P.; writing – review, all authors.

## Supporting information


**Figure S1:** (A) Spectral irradiance of the UV/VIS lamp. (B) Spectral characteristics of long‐pass edge filter passing wavelengths above 500 nm (C) Spectral characteristics of two combined filters comprising a long‐pass edge filter passing wavelengths above 350 nm and a short‐pass edge filter passing wavelengths above 500 nm.
**Figure S2:** Comparison of the spectral distribution of the solar spectrum (top) and data from an older product, Hamamatsu LightningCure spotlight source LC8‐01 with L8251 lamp used in this study (bottom); however, the current product is LC8‐01A with L10852. The solar spectral distribution covers a broad continuous range from UV to infrared, while the LC8‐01 lamp exhibits characteristic emission peaks in the UV region. These spectra illustrate the differences between natural solar irradiation and the artificial light source employed in the experiments.

## Data Availability

The data that support the findings of this study are available on request from the corresponding author. The data are not publicly available due to privacy or ethical restrictions.
