# Supplementary material for: Chemiexcitation in Ex Vivo Porcine Skin Model
Source: Pigment Cell Melanoma Res. 2025 Oct 14;38(6):e70060. doi: 10.1111/pcmr.70060 (PMC12521798; doi:10.1111/pcmr.70060)
Supplement: Supplementary file 1 — Figure S1: (A) Spectral irradiance of the UV/VIS lamp. (B) Spectral characteristics of long‐pass edge filter passing wavelengths above 500 nm (C) Spectral characteristics of two combined filters comprising a long‐pass edge filter passing wavelengths above 350 nm and a short‐pass edge filter passing wavelengths above 500 nm. Figure S2: Comparison of the spectral distribution of the solar spectrum (top) and data from an older product, Hamamatsu LightningCure spotlight source LC8‐01 with L8251 lamp used in this study (bottom); however, the current product is LC8‐01A with L10852. The solar spectral distribution covers a broad continuous range from UV to infrared, while the LC8‐01 lamp exhibits characteristic emission peaks in the UV region. These spectra illustrate the differences between natural solar irradiation and the artificial light source employed in the experiments. [file PCMR-38-0-s001.docx]

**Chemiexcitation in ex vivo porcine skin model**

Pavel Pospíšil*, Vendula Paculová, Ankush Prasad, Michal Berecka

Department of Biophysics, Faculty of Science, Palacký University, Šlechtitelů 27, Olomouc, Czech Republic

*Corresponding author: [pavel.pospisil@upol.cz](mailto:pavel.pospisil@upol.cz)

Supplementary figures

Figure S1: (A) Spectral irradiance of the UV/VIS lamp. (B) Spectral characteristics of long-pass edge filter passing wavelengths above 500 nm (C) Spectral characteristics of two combined filters comprising a long-pass edge filter passing wavelengths above 350 nm and a short-pass edge filter passing wavelengths above 500 nm.


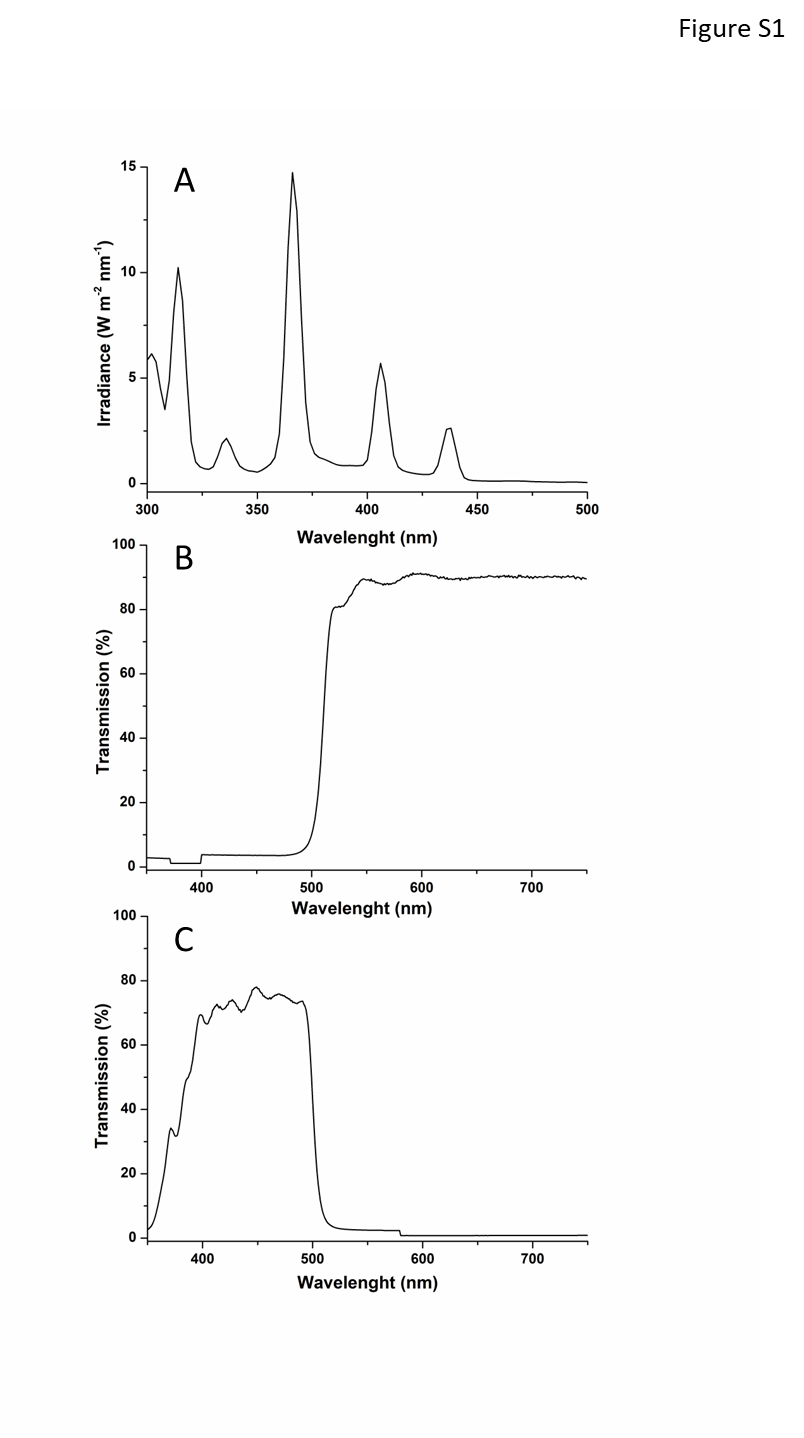


Figure S2. Comparison of the spectral distribution of the solar spectrum (top) and data from an older product, Hamamatsu LightningCure spotlight source LC8-01 with L8251 lamp used in this study (bottom); however, the current product is LC8-01A with L10852. The solar spectral distribution covers a broad continuous range from UV to infrared, while the LC8-01 lamp exhibits characteristic emission peaks in the UV region. These spectra illustrate the differences between natural solar irradiation and the artificial light source employed in the experiments.


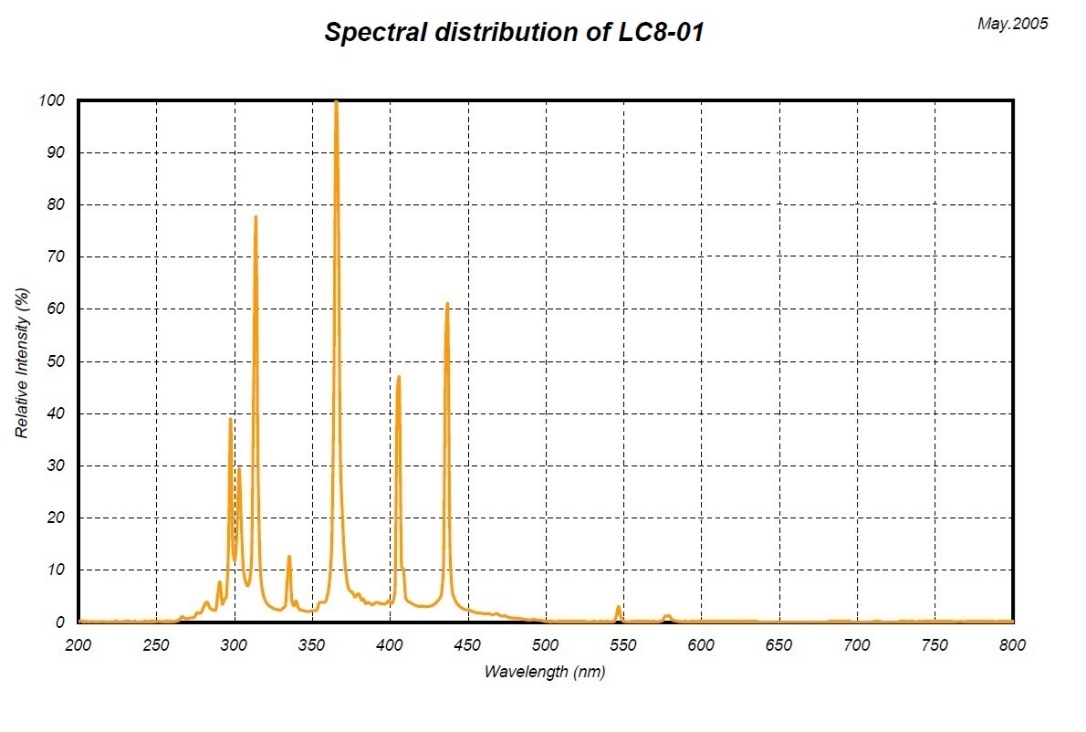


*relative intensity [%]*

*wavelength [nm]*
